# An Integrated Strategy for Analyzing the Unique Developmental Programs of Different Myoblast Subtypes

**DOI:** 10.1371/journal.pgen.0020016

**Published:** 2006-02-17

**Authors:** Beatriz Estrada, Sung E Choe, Stephen S Gisselbrecht, Sebastien Michaud, Lakshmi Raj, Brian W Busser, Marc S Halfon, George M Church, Alan M Michelson

**Affiliations:** 1 Division of Genetics, Department of Medicine, Brigham and Women's Hospital and Harvard Medical School, Boston, Massachusetts, United States of America; 2 Howard Hughes Medical Institute, Boston, Massachusetts, United States of America; 3 Department of Genetics, Harvard Medical School, Boston, Massachusetts, United States of America; North Carolina State University, United States of America

## Abstract

An important but largely unmet challenge in understanding the mechanisms that govern the formation of specific organs is to decipher the complex and dynamic genetic programs exhibited by the diversity of cell types within the tissue of interest. Here, we use an integrated genetic, genomic, and computational strategy to comprehensively determine the molecular identities of distinct myoblast subpopulations within the *Drosophila* embryonic mesoderm at the time that cell fates are initially specified. A compendium of gene expression profiles was generated for primary mesodermal cells purified by flow cytometry from appropriately staged wild-type embryos and from 12 genotypes in which myogenesis was selectively and predictably perturbed. A statistical meta-analysis of these pooled datasets—based on expected trends in gene expression and on the relative contribution of each genotype to the detection of known muscle genes—provisionally assigned hundreds of differentially expressed genes to particular myoblast subtypes. Whole embryo in situ hybridizations were then used to validate the majority of these predictions, thereby enabling true-positive detection rates to be estimated for the microarray data. This combined analysis reveals that myoblasts exhibit much greater gene expression heterogeneity and overall complexity than was previously appreciated. Moreover, it implicates the involvement of large numbers of uncharacterized, differentially expressed genes in myogenic specification and subsequent morphogenesis. These findings also underscore a requirement for considerable regulatory specificity for generating diverse myoblast identities. Finally, to illustrate how the developmental functions of newly identified myoblast genes can be efficiently surveyed, a rapid RNA interference assay that can be scored in living embryos was developed and applied to selected genes. This integrated strategy for examining embryonic gene expression and function provides a substantially expanded framework for further studies of this model developmental system.

## Synopsis

Animal development requires cells in complex organs to acquire distinct identities. During the development of the body wall musculature of the fruit fly, a pool of apparently identical cells gives rise to two types of muscle precursors, both of which are required for the appearance of functioning muscles. These identities depend on broad programs of gene expression. The authors attempt to dissect the complements of expressed genes that define these two different cell types by integrating modern methods in genetics, genomics, and informatics. By purifying informative cells from normal embryos and mutants that perturb muscle development, assaying their genomewide gene expression programs, and combining experiments statistically, they have identified fivefold more founder-specific genes than were previously suspected to characterize this cell type. The expression patterns of hundreds of genes were examined in whole embryos to test the statistical predictions, permitting the authors to estimate how many more cell type–specific genes remain to be discovered. Finally, dozens of the genes highlighted by these methods were tested for direct involvement in muscle development, and several new players in this process are reported. The integrated strategy used here can be generalized for studying genetic programs in other complex tissues.

## Introduction

Transcriptional regulation plays a central role in metazoan development by establishing cell-specific patterns of gene expression that represent coordinate responses to extrinsic signals and intrinsic programming [1,2]. Thus, detailed knowledge of the genes that are spatially and temporally coexpressed at the cellular level in a particular developmental context will not only provide insight into the logic of transcriptional networks but also define the downstream effectors of morphogenesis. Given the cellular diversity present in most tissues, it would be ideal to derive the entire genetic program of each individual cell type and to determine the response of each differentially expressed gene to perturbations of the pathways that regulate formation of that organ. Defining such cell-specific gene expression signatures and mapping the sequential steps involved in their generation are both essential to achieving a systems-level view of development [3,4].

Traditional studies have monitored only one or a few cell-type specific markers at a time using different genetic backgrounds to perturb the developmental process of interest. In many cases, such approaches have yielded sets of regulatory inputs and responses that provide the conceptual underpinnings for considering development in the broader terms of component interactions and network architecture [5,6]. However, to test the generality of hypotheses derived from the study of small numbers of genes, it is essential to acquire a comprehensive assessment of the gene expression changes occurring in response to a known set of developmental regulators.

Elaborating an integrated and systematic experimental approach to identify and functionally characterize such genes and their *cis*-regulatory sequences in a metazoan model organism remains a significant and largely unsolved challenge. In yeast, pooled expression profiles derived for multiple genotypes and chemical treatments have proved extremely valuable for dissecting biological pathways [7]. In principle, it should be possible to generate equally illuminating expression profile compendia for the development of multicellular organisms. Large numbers of datasets have been combined in a few cases for this purpose [8,9], but these studies did not focus on a particular aspect of development. Here, we have used such a comprehensive approach to examine the molecular identities of myoblast subtypes in the *Drosophila* embryo, results that yield new information about the composition of the muscle regulatory network.

Myogenesis initiates with the segregation of two types of myoblasts from the somatic mesoderm: founder cells (FCs) and fusion-competent myoblasts (FCMs) [10]. Each FC possesses a unique identity and seeds the formation of an individual myotube by fusing with the more homogeneous population of FCMs. Of the known early muscle-specific genes, some are specific to only one myoblast type, while others are expressed in both. Many of these genes encode transcription factors that are essential for myoblast specification [11–16]. Intercellular signals act in different combinations to promote the formation of FCs and FCMs [10,17]. This process is best understood for a subset of FCs that express *even skipped (eve)* [18–21]. Wingless (Wg, a Wnt family member) and Decapentaplegic (Dpp, a member of the bone morphogenetic protein superfamily) first cooperate to render a large domain of mesodermal cells competent to respond to a subsequent inductive signal mediated by two receptor tyrosine kinases (RTKs), an epidermal growth factor (EGF) receptor (EGFR) and the fibroblast growth factor (FGF) receptor (FGFR) encoded by *heartless (htl).* Localized RTK activation within the competence domain stimulates the Ras pathway and the formation of Eve-expressing equivalence groups [18]. Lateral inhibitory signaling by Notch then allows a single Eve progenitor to emerge from each equivalence group under the continued influence of Ras [19], with the remaining Notch-inhibited cells assuming an FCM identity characterized by expression of *lame duck (lmd)* [14–16]. Since FCs are derived by the asymmetric division of progenitors [22,23], the Ras pathway favors FC formation, while Notch promotes FCM development from mesodermal equivalence groups.

Integration of the Wg, Dpp, and Ras pathways occurs through the direct convergent regulation of *eve* by the three corresponding signal-activated transcription factors bound to a specific enhancer in the context of two mesodermal selectors [24–26]. Thus, distinct myoblast identity codes are generated by the combinatorial functions of Wg, Dpp, EGF, FGF, and Notch signals. These signaling codes are in turn mirrored in transcriptional codes that induce the changes in gene expression that are characteristic of individual FCs and FCMs. Collectively, this knowledge provides the logical foundation for genomic and computational investigations of muscle gene transcriptional regulation in the *Drosophila* embryo.

Gene expression profiling of the *Drosophila* embryonic mesoderm has been undertaken in several prior studies. In one approach, mutations in early dorsoventral patterning genes were used to eliminate or overproduce mesodermal cells, and genes whose expression is enriched in the mesoderm were identified by microarray analysis [14,27]. A modification of this approach in which the Ras or Notch pathway was constitutively activated in a *Toll^10b^* mutant—a genetic background that drastically disrupts gastrulation and converts the entire embryo to mesoderm—led to the identification of a small number of genes that are specific to FCs or FCMs [28]. However, the latter study was limited by several factors, including the complete lack of inductive ectoderm and its differentiated derivatives in *Toll^10b^* embryos, the absence of Dpp in these embryos, the disruption of normal cellular interactions within the overproduced mesoderm, independent validation of only a few microarray predictions so that a true-positive detection rate could not be reliably estimated, and the use of a cDNA microarray that represented only 40% of the genes in the entire *Drosophila* genome. It is likely, therefore, that many more FC and FCM genes remain to be discovered.

To address this question, we designed a different strategy for analyzing cell type–specific genetic programs for a complex tissue that circumvents the previously encountered difficulties and is more generally applicable. This approach integrates genetic perturbations of development, purification of primary embryonic cells of interest, microarray-based genomewide transcriptional profiling, statistical meta-analysis of the pooled gene expression datasets, and large-scale validation by in situ hybridization of gene expression patterns predicted by the computational analysis. Applying this strategy, we identified and validated several hundred genes that are uniquely expressed in FCs, FCMs, or both myoblast types. Finally, we used in vivo RNA interference (RNAi) to rapidly assess the myogenic functions of several newly identified myoblast genes. In a separate but complementary effort, information derived from the present studies was applied to a new computational method for analyzing the relative contribution of individual transcription factor binding sites to combinatorial transcriptional codes (A. A. Philippakis, B. Busser, S. S. Gisselbrecht, F. S. He, B. Estrada, A. M. Michelson, and M. L. Bulyk, unpublished data). Taken together, the systematic strategy used here provides significant new insights into embryonic myogenesis and represents an integrated experimental framework that can be applied to related investigations in other developmental contexts.

## Results

### Purification of Mesodermal Cells by Flow Cytometry

**Figure 1:**
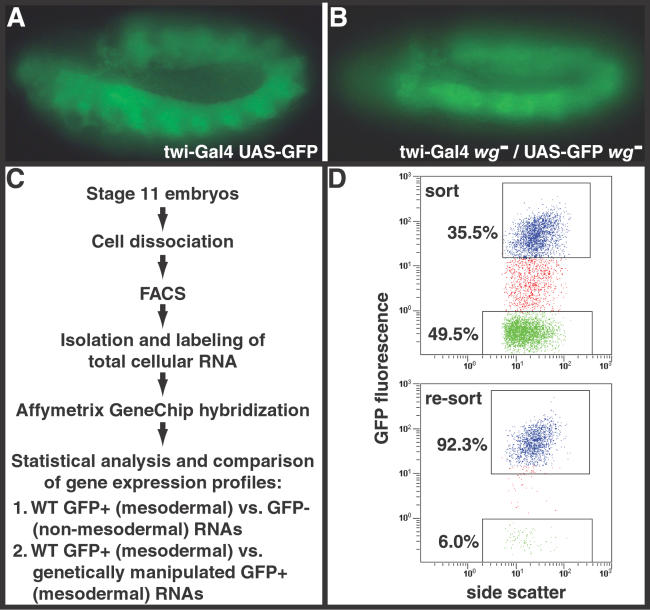
Experimental Strategy for Transcriptional Profiling of Purified Embryonic Mesodermal Cells (A) Embryos transgenic for *Gal4* under the control of the *twi* promoter and *GFP* under UAS control express GFP specifically in mesodermal cells. (B) If *twi-Gal4* and UAS-*GFP* transgenes are maintained on mutant chromosomes, only homozygous mutant mesoderm expresses GFP when the resulting flies are mated. (C) Overview of the workflow to obtain genomewide expression information on mesodermally enriched and genotype-responsive genes. (D) A representative FACS experiment, showing initial sorting of all cells from wild-type embryos (top; upper box represents GFP-positive sort window [blue], lower box represents GFP-negative sort window [green]) and resorting of the purified cell population (bottom). Sorting parameters routinely achieved greater than 90% pure GFP-positive cells.

To increase the sensitivity of detecting myoblast transcripts in microarray expression profiling experiments, we first developed a method to purify both wild-type and mutant cells of interest from whole *Drosophila* embryos. Green fluorescent protein (GFP) was targeted to the mesoderm using the *Gal4*-UAS technique, with *twi*-*Gal4* as a specific driver and a UAS-*GFP* transgene as the reporter (Figure 1A) [29,30]. We used the binary nature of this expression system to target GFP not only in a tissue-specific manner but also such that only mutant cells would be labeled for any loss-of-function genotype. This goal was accomplished by recombining the *twi*-*Gal4* construct onto a selected mutant chromosome in one strain and the UAS-*GFP* reporter onto the same mutant chromosome in a second strain. Crossing these two strains results in GFP expression only in mutant mesodermal cells; neither wild-type mesoderm nor mutant nonmesodermal cells express GFP in progeny embryos (Figure 1B). Similarly, it is possible to introduce a second UAS transgene that encodes a constitutively activated or dominant negative form of a signal transduction component or transcription factor as another means of perturbing normal development [19,24]. Most important, selection of an appropriate combination of specific *Gal4* lines and additional genetic backgrounds enables this strategy to be targeted to the development of any tissue or cell type.

Embryos were collected, incubated to the stage during which FCs and FCMs are specified, and then gently dissociated to yield a single cell suspension. GFP-expressing and non–GFP-expressing cells were separated by fluorescence activated cell sorting (FACS), total cellular RNA was isolated from each population, and the RNA was labeled for hybridization to Affymetrix GeneChip arrays (Figure 1C). A representative flow cytometry scatterplot for purification of wild-type mesodermal cells is illustrated in Figure 1D. Cell-sorting parameters were optimized for achieving greater than 90% cell purity in all experiments.

### Identification of Genes with Enriched Expression in Wild-Type Mesodermal Cells

We first compared the RNA profiles for GFP-positive versus GFP-negative cells purified from wild-type embryos. Using the statistical methods detailed in Protocol S1, Analysis Method A, 335 probe sets were identified to have higher expression levels in GFP-positive cells than in the rest of the embryo. Of these, approximately 200 had not previously been described as having mesodermal expression. To validate these results, we undertook in situ hybridizations in wild-type embryos using probes corresponding to 207 genes enriched in the GFP-positive population (including some that had been described previously but had not been extensively characterized). Combining these results with data from the literature, we calculated a true-positive detection rate of 95.3% for genes enriched in GFP-expressing cells. Genes expressed in a wide variety of mesodermal derivatives were identified, including somatic and visceral muscle precursors, fat body, hemocytes, and heart (Figure S1 and Table S1). Having established the feasibility of expression profiling FACS-purified mesodermal cells, further experiments were designed to more completely characterize the expression programs of different myoblast subpopulations.

### Prediction of Candidate Myoblast-Specific Genes from a Compendium of Mesodermal Gene Expression Profiles

**Figure 2:**
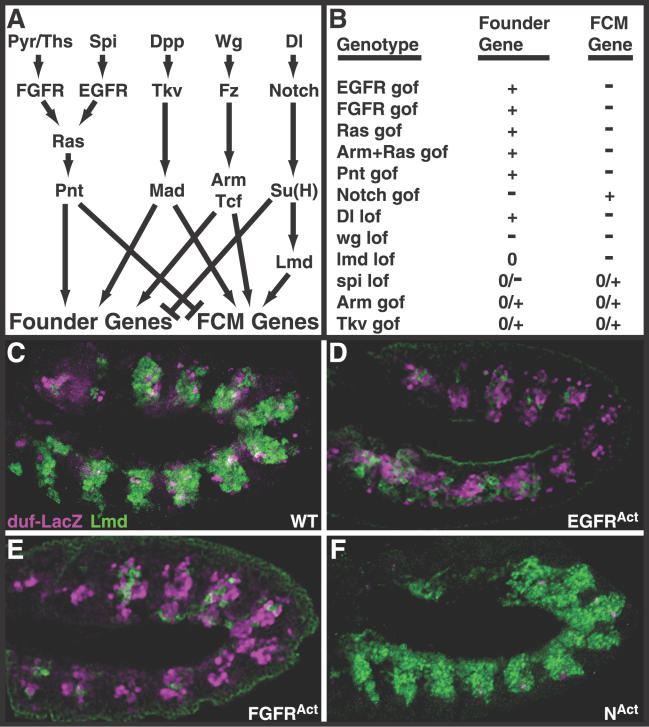
Differential Genetic Inputs to Two Classes of Somatic Myoblasts (A) A network of signaling molecules and transcription factors is known to positively and negatively regulate the specification of muscle FCs and FCMs. (B) Predicted behavior of genes specific to these cell types when key components of the network are genetically perturbed (+ indicates increased mesodermal expression relative to wild-type; −, decreased expression; 0, no change). (C–F) Expression of an FC marker (*duf-lacZ,* magenta) and an FCM marker (Lmd, green) show that in wild-type somatic mesoderm (C), FCs comprise a small number of individual cells, and the remainder of somatic myoblasts are FCMs. Constitutive activation of the EGF receptor (D) or FGF receptor (E) greatly expands the FC population at the expense of FCMs; remnant Lmd protein is excluded from nuclei. Conversely, constitutive activation of Notch signaling (F) largely eliminates the FC population with either no effect or perhaps a slight increase in FCMs apparent at the indicated resolution.

A key feature of our experimental strategy is the use of specific genetic backgrounds to selectively perturb gene expression based on existing knowledge of relevant developmental pathways. The intercellular signaling network involved in *Drosophila* FC and FCM development is shown in Figure 2 [10,17]. In the few examples studied at single cell resolution, the RTK/Ras pathway was found to induce FC identities, whereas Notch had a similar function for FCMs [14–16,18,19,31]. To assess whether these two signals are differentially involved in the specification of all somatic myoblasts, we used a *dumbfounded (duf)* enhancer trap line as a global FC marker [32,33], and an antibody directed against Lmd as a marker of all FCMs [15] (Figure 2C). Mesodermal expression of either constitutively activated EGFR or FGFR had the same effect: FCs were markedly overproduced at the expense of FCMs in all regions of the somatic mesoderm (Figure 2D and 2E). Conversely, Notch activation blocked formation of most, if not all, FCs, with either no effect or perhaps a slight increase in FCMs (Figure 2F). Thus, the EGFR/FGFR and Notch pathways have opposing effects on the determination of virtually all FCs and FCMs. Given these results, we predicted that loss- and gain-of-function genetic manipulations of these pathways would generate global changes in myoblast-specific gene expression, as indicated in Figure 2B, and that these patterns should facilitate the rapid categorization of FC and FCM genes on a genomewide scale.

**Figure 3:**
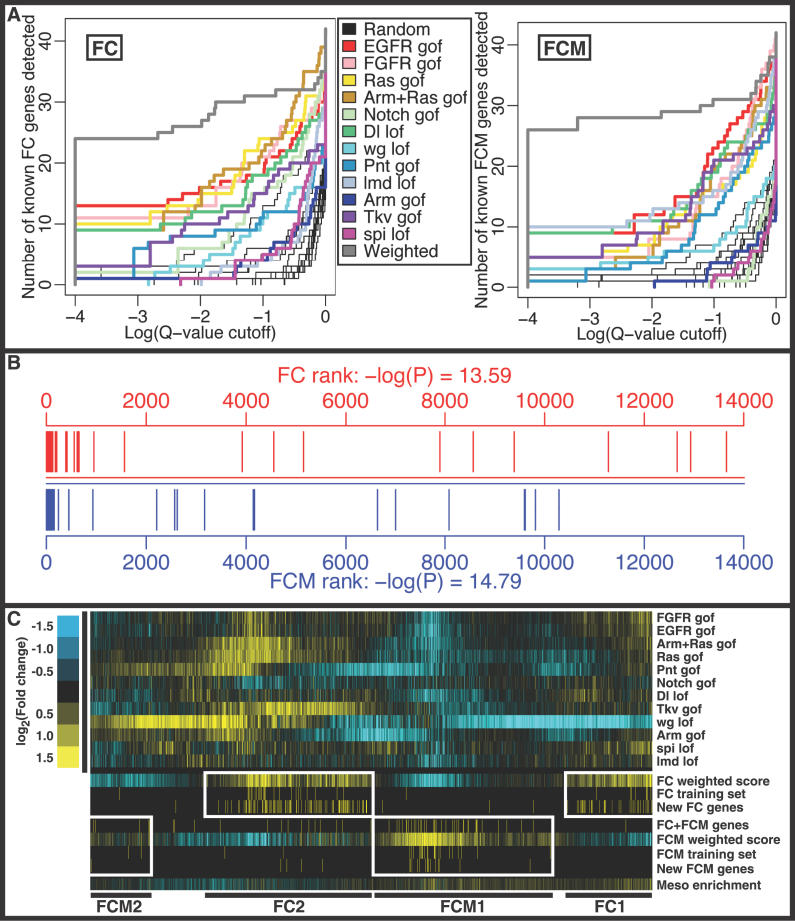
Statistical Meta-Analysis of an Expression Profiling Compendium for Predicting Myoblast-Specific Genes (A) Detection curves showing the number of probe sets from the training set detected, as a function of q-value, for FC genes (left) and FCM genes (right). In each panel, the predictive value of individual genotype/wild-type comparisons (various colors; see legend) are compared to randomly generated rankings (thin black lines) and to composite rankings derived from a weighted combination of all datasets (gray). To avoid introducing biases for or against any genotype, the training sets were composed of known genes from the literature as well as the mesodermally enriched genes that had been verified by in situ hybridization in this study to be FC or FCM genes, for a total of 43 FC probe sets and 42 FCM probe sets (Table S2). (B) All probe sets on the chip were ranked according to their degree of FC-like (red axis) or FCM-like (blue axis) expression pattern, using their weighted T-scores. The ranks of the training set probe sets (FC in red, FCM in blue) within their respective axes are plotted as thin vertical lines, revealing the extent to which optimization concentrates each training set at the top of its corresponding rank. *P* values shown are from the Wilcoxon-Mann-Whitney *U* test. Thick lines reflect the merging of individual thin vertical lines. (C) Clustering of genotype data (first 12 rows) by self-organizing maps produces two clusters enriched for known and predicted FC genes (FC1 and FC2, white boxes) and two clusters enriched for known and predicted FCM genes (FCM1 and FCM2, white boxes). For reference, FC- and FCM-weighted T-scores are depicted (yellow: T > 0, blue: T < 0, and saturated yellow/blue colors correspond to the 98% percentile of the absolute T-values). Also highlighted are the locations of validated FC and FCM genes (yellow lines), as well as mesodermal fold change levels (“meso enrich”).

A compendium of gene expression profiles specifically targeted to muscle development was generated for mesodermal cells purified from 12 genetic backgrounds (Figure 2B). A meta-analysis was then designed to optimize the assignment of genes to one or the other myoblast category based on each gene's collective behavior in the expression profile compendium. For example, any gene that is upregulated relative to wild-type in RTK/Ras, Dpp, or Wg pathway-activating conditions, upregulated in a *Dl* mutant, downregulated with Notch activation, and downregulated in a *wg* mutant should have a high probability of being expressed in muscle FCs. Of note, any one genotype alone detected less than 40% of known FC genes and less than 30% of known FCM genes (at q = 0.01; Figure 3A), suggesting that many more genes that are specifically transcribed in each of these cell types remain to be identified. We therefore factored into the meta-analysis not only the expected trends in gene expression for each genetic manipulation but also a weight factor that reflects the relative contribution of each genotype to the detection of known myoblast-specific genes (Protocol S1, Analysis Method E).

To score the genes with respect to FC- or FCM-like expression response, we used a statistical metric (“T”) [34], which is a weighted sum of the *t*-statistics from each genotype versus wild-type comparison (Protocol S1, Analysis Method E). The weights in this sum were optimized to account for the differential sensitivity of the genotypes in detecting training sets of FC or FCM genes (Figure 3A). To avoid introducing biases for or against any genotype, these training sets primarily contained the mesodermally enriched genes that had been verified by in situ hybridization in this study to be FC or FCM genes, as well as known genes of each class taken from the literature, for a total of 43 FC probe sets and 42 FCM probe sets (Table S2). Clear distinctions exist between the optimized weight profiles derived for FC and FCM genes (Figure S2A and S2C), consistent with each genotype differentially affecting gene expression in the two myoblast types. Using these two sets of weights, we then calculated two T-scores for every gene, one representing FC-like and the other, FCM-like, expression responses.

When all genes were ordered based on their FC and FCM T-scores, both training sets were preferentially located at the tops of their respective ranks (*P* < 10^−13^ for FC genes and *P* < 10^−14^ for FCM genes, using the Wilcoxon-Mann-Whitney *U* test; Table S2 and Figure 3B). We also were able to assign significance level estimates to the T-scores by applying random permutations to the expression datasets. These calculations yielded a q-value for each gene, which is the predicted false-positive fraction (number false positive/number called positive) when using that gene's T-score as the cutoff for significance [35]. Figure 3A shows the improved sensitivity achieved by our meta-analysis for the detection of FC and FCM genes. When combining multiple datasets, we were able to detect more known FC and FCM genes at a given q-value than when using any genotype individually. This outcome is not entirely the result of simply having more replicates, since the efficacy of the meta-analysis also benefits from the inclusion of related results from multiple genotypes that independently and differentially perturb the developmental process of interest (see Discussion).

From the targeted expression profile compendium, we predicted a total of 373 (q = 0.002) and 276 (q = 0.002) genes with FC- and FCM-like responses, respectively (Protocol S1, Analysis Method F; Figure S2B and S2D). After extensive follow-up using in situ hybridization, lists of validated FC, FCM, or FC + FCM genes were then queried for relative enrichment of Gene Ontology (GO) terms (Table S3). For FC genes, overrepresented molecular function categories include transcriptional regulation, transmembrane receptor protein kinase activity, cytoskeletal protein binding, and small GTPase regulatory/interacting proteins, with enrichment for biological processes such as cell surface receptor–linked signal transduction, cell adhesion, cell motility, small GTPase mediated signal transduction, and mesoderm cell fate specification. In contrast, the validated FC + FCM gene candidates are biased toward ribosome and protein biosynthesis. There were too few validated FCM genes to yield many statistically enriched GO terms, but the two that passed our cutoff criteria were muscle and mesoderm development.

We next clustered the expression profiling data derived for all genotypes and found that both the training sets and subsequently identified FC and FCM genes segregate into two broad subclusters for each cell type (Figure 3C). FC1 genes largely follow the expected responses to the set of multifactorial genetic perturbations (Figure 2B), whereas FC2 genes have an unanticipated response to *wg* loss-of-function (increased expression) and a stronger than expected Dpp gain-of-function response. Such an aberrant *wg* effect can occur for somatic FC genes that are also expressed in the visceral mesoderm, which is expanded in *wg* mutant embryos [36,37]. Known FCM genes are predominantly located in subcluster FCM1, in agreement with the canonical FCM expression pattern (Figure 2B).

### Validation of Results Derived from the Targeted Expression Profile Compendium

**Figure 4:**
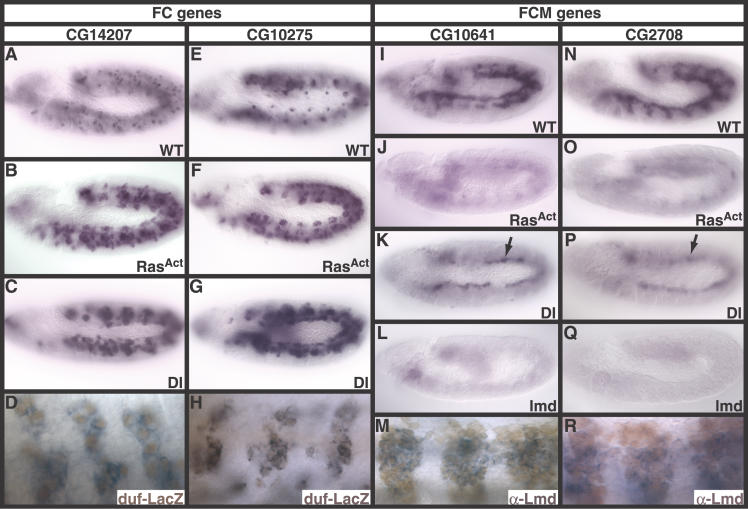
Empirical Validation of Predicted FC and FCM Genes CG14207 (A–D) and CG10275 (E–H) are representative FC genes identified in the present work with meta-analysis ranks of 6 and 11, respectively (Figure 3B and Table S1). RNA in situ hybridization shows that each is normally expressed in a characteristic subset of somatic myoblasts (A and E) and in an expanded population in embryos in which Ras is ectopically activated (B and F) or that are mutant for *Dl* (C and G). Staining of in situ–hybridized embryos with the founder marker *duf-lacZ* (orange nuclei: D and H) reveals extensive coexpression. CG10641 (I–M) and CG2708 (N–R) are representative FCM genes ranked in the meta-analysis as 38 and 23, respectively. In wild-type embryos (I and N), they are expressed in the majority of somatic and visceral myoblasts. This expression is largely lost in activated Ras embryos (J and O) and in the somatic mesoderm of *Dl* mutant embryos (K and P), although the latter retain expression in the visceral mesoderm (arrows). Expression of these genes is lost in *lmd* mutant embryos (L and Q), and is normally restricted to a subset of Lmd-expressing cells (orange nuclei: M and R).

To validate microarray meta-analysis predictions, in situ hybridizations were performed for large numbers of genes using embryos with informative genotypes. For example, since Ras gain-of-function and *Dl* loss-of-function overproduce FCs at the expense of FCMs [18,19,38,39], a gene specifically expressed in FCMs or FCs should have reduced or increased expression, respectively, in these genetic backgrounds (Figure 4). Moreover, newly identified FC genes coexpress *duf,* an established FC marker [33] (Figure 4D and 4H), while predicted FCM genes coexpress the known FCM gene, *lmd* [14–16] (Figure 4M and 4R).

To assess the accuracy of the meta-analysis, we examined how many true positives are found among the genes highly ranked as being expressed in each type of myoblast (Table S2). Of 213 randomly selected genes from among the top-ranked 373 FC candidates, 118 (55%) were validated as authentic FC genes, that is, actually expressed in founder cells by embryonic in situ hybridizations in the above-mentioned genetic backgrounds. When 123 of the predicted 276 FCM genes were similarly examined by in situ hybridization, 18 (15%) were found to have FCM-specific expression patterns, while an additional 40 (33%) were found to be expressed in both FCs and FCMs. Taken together, these findings suggest that, while FC gene predictions derived from the present experimental design are very accurate, the hypothesized specificity of the genetic manipulations for FCM genes is confounded by genes that are expressed in both myoblast types. Of note, this conclusion could only be derived from the large-scale in situ hybridization data obtained here, experiments that have not frequently been undertaken in other transcriptional profiling studies to validate microarray results. Using the present findings, it is apparent that a previous microarray-based study also had a significant false-positive rate of FCM gene prediction, although the authentic FC gene discovery rate in that case was comparably high. However, it is important to note that significantly fewer total gene numbers were detected in the earlier study for both myoblast classes [28] (see Table S1 for details). Pooling all of the currently available data, 160 FC and 51 FCM genes are known, of which 131 and 45, respectively, were identified and validated in the present studies. Extrapolating from our findings, we estimate that FCs and FCMs actually express a total of about 321 and 82 unique genes, respectively (see Protocol S1, Analysis Method F).

### Differential Regulation of FCM Genes by the Zinc Finger Transcription Factor, Lmd

**Figure 5:**
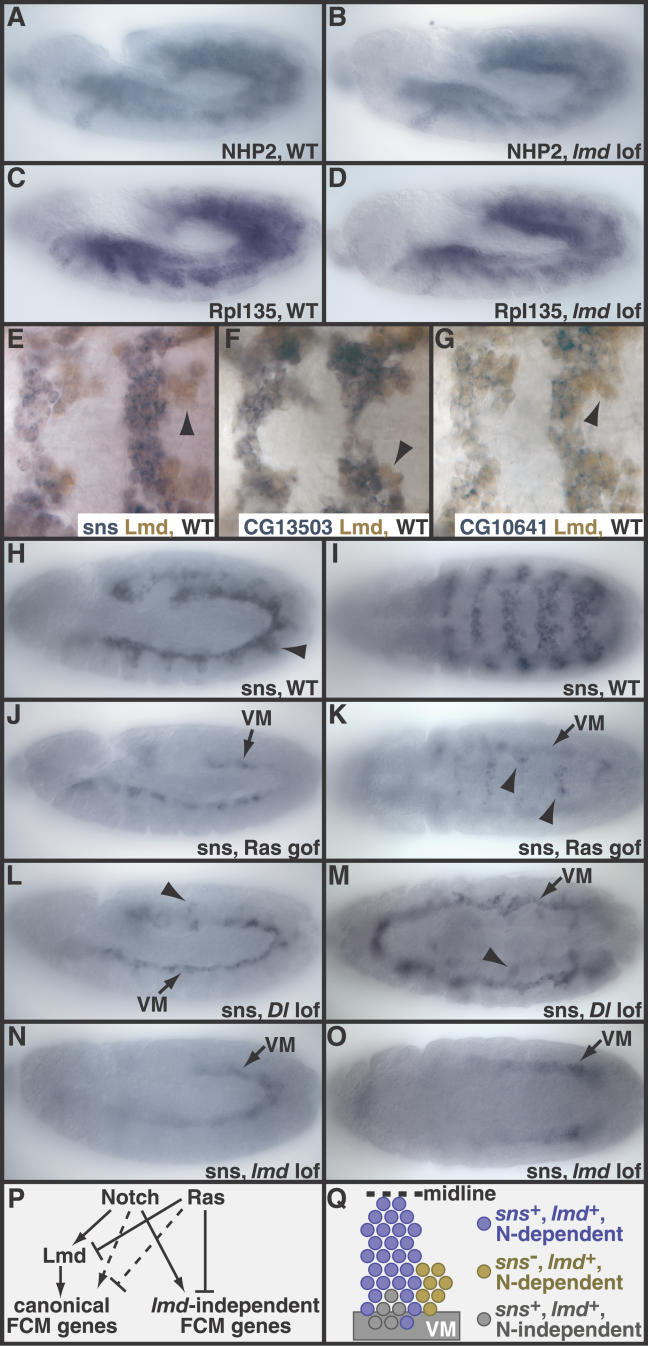
Heterogeneity in the Regulation of FCM Gene Expression (A–D) In situ hybridization of wild type and *lmd* mutant embryos with NHP2 (A and B) and RpI135 (C and D) gene probes shows that expression of these two FCM genes is independent of Lmd regulation. (E–G) Subset of ventral Lmd-expressing myoblasts (orange nuclei) without the expression of FCM markers such as *sns*, CG13503, and CG10641 (arrowheads in E, F, and G, respectively). (H–O) A subset of ventral myoblasts displays unexpected behavior. Wild-type expression of *sns* is observed in lateral (H) and ventral (I) somatic myoblasts (arrowhead in H). Lateral somatic myoblast expression is uniformly downregulated in Ras gof (J), *Dl* mutant (L), and *lmd* mutant (N) embryos, although visceral mesoderm (VM) expression is largely unchanged. A small ventral population of *sns*-expressing myoblasts remains in embryos with constitutive Ras activation (K, arrowheads) or loss of *Dl* (M), but expression in these cells is lost in *lmd* mutant embryos (O). (P) Expression of previously described (“canonical”) FCM-specific genes depends on Lmd, which is activated by Notch signaling and repressed by Ras signaling; a subset of FCM genes (A–D) are under the control of the Ras and Notch signaling pathways but do not require Lmd for their expression. (Q) The majority of somatic FCMs (blue) express *sns* and *lmd* and require Notch signaling for their specification; we have identified a subpopulation of cells (brown) that express *lmd* but not *sns* or other FCM markers (E–G) and additional cells (gray, including VM) that express *sns* even in the absence of the Notch ligand *Dl.*

Expression of the vast majority of newly identified FCM genes requires *lmd,* which encodes a transcription factor that is essential for FCM development [14–16] (Figure 4L and 4Q and Table S1). However, four of the validated FCM-specific genes (Figure 5) were unexpectedly found to be independent of Lmd for their expression (Figure 5A–5D and Table S1). Further analysis revealed many genes that in general behave like FCM genes but actually exhibit more complex region-specific expression patterns. For example, some genes are *lmd* dependent in dorsal and lateral regions of the embryo (Figure 5H, 5J, 5L, and 5N and data not shown) but have a ventral expression domain that does not include all Lmd-positive myoblasts (Figure 5E–5G). Furthermore, expression of these latter genes in some ventral myoblasts responds to both Ras activation and loss of *Dl* function in a manner akin to FC rather than FCM genes (Figure 5I, 5K, and 5M), although they are entirely FCM like in their dependence on *lmd* (Figure 5O). In some but not all cases, genes expressed in the somatic mesoderm that are *lmd* dependent do not require *lmd* for their expression in the visceral mesoderm (compare Figures 4L and 4Q and Figures 5N and 5O; Table S1), underscoring the differential response of such genes to loss of *Dl* function in these two mesodermal subdivisions (Figure 4K and 4P). These findings are summarized in Figure 5P and 5Q.

### Functional Analysis of Newly Identified Myoblast Genes

**Figure 6:**
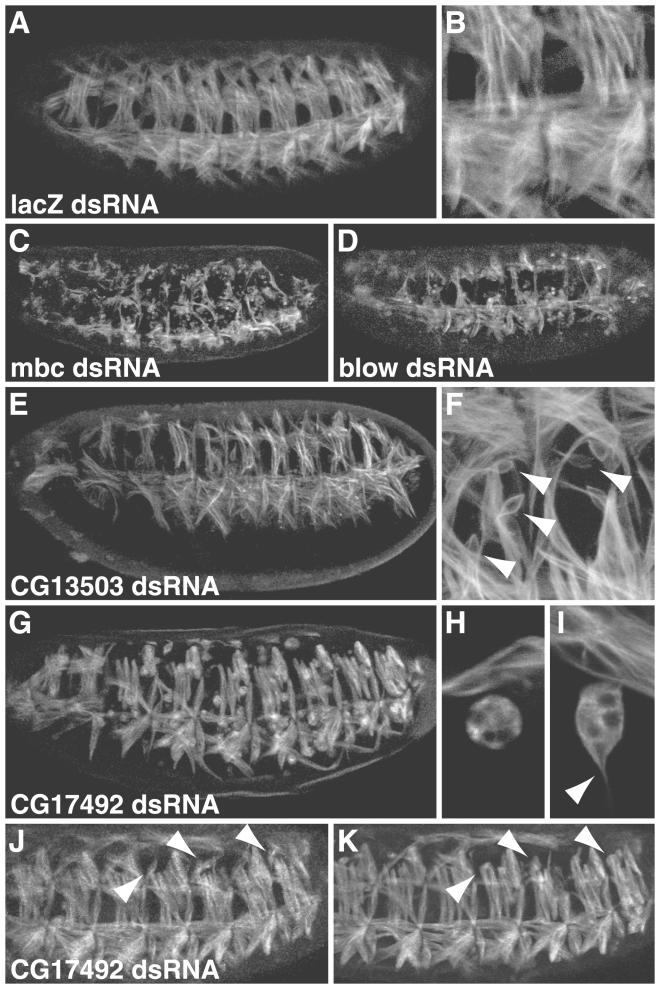
RNAi Analysis of Selected Myoblast Genes (A and B) Live embryos expressing a tau-GFP fusion protein under control of the myosin heavy chain promoter and injected with an inactive control double-stranded *lacZ* RNA have a wild-type mature muscle pattern. Note, at high magnification (B), the complete absence of unfused myoblasts at this stage. (C and D) Injection of dsRNA for the known muscle fusion genes *mbc* (C) and *blow* (D) phenocopy mutations in these genes. (E and F) RNAi directed against the FCM gene CG13503 causes an overall reduction and disorganization of muscle fibers (E), with persistence of unfused myoblasts (arrowheads in F), consistent with a fusion defect. (G–K) Injection of dsRNA for the FC gene CG17492 results in the formation of multinucleate myospheres from only certain muscle fibers. A severely affected embryo (G) demonstrates the complete sparing of certain muscle groups, while other muscles appear as spheres (H) or as compact masses with thin extensions (arrowhead, I). While some abnormalities are apparent before any muscle contraction is visible (J), the same embryo observed later (K) shows that some muscles that had appeared morphologically normal have now formed myospheres (compare arrowheads in J and K).

To screen for the developmental functions of newly identified myoblast genes, we modified a whole embryo RNAi assay [40] to permit the rapid scoring of muscle patterning phenotypes. Double-stranded RNAs (dsRNAs) were injected into blastoderm embryos expressing a tau-GFP fusion protein under myosin promoter control, which enables the complete muscle pattern to be visualized after the embryos develop [41] (Figure 6A and 6B). Injection of dsRNAs corresponding to genes with known myogenic functions phenocopied their genetic loss-of-function with complete penetrance, while a nonspecific dsRNA had no effect [42–44] (Figure 6A–6D). Since this assay involves a 1-d turnaround without further embryo manipulation, multiple genes can be screened simultaneously.

Selected RNAi results are shown in Figure 6E through 6K. Injection of dsRNA derived from CG13503—an FCM-specific gene that encodes verprolin, an actin binding protein—causes a reduction in myoblast fusion (Figure 6E and 6F). Based on the presence of single, unfused muscle cells in these embryos, we have named CG13503 “*solas”* (*sola* means “alone”). RNAi for CG17492—an FC-specific gene whose mammalian ortholog is skeletrophin [45]—causes a more severe loss of normal myofibers and their replacement by multinucleated myospheres, some of which extend short processes (Figure 6G–6I). This phenotype is observed prior to the onset of muscle contraction—which can be directly visualized in living embryos—yet it becomes progressively more severe as the muscles begin to contract (Figure 6J and 6K). The association of unattached myospheres with the effects of CG17492 RNAi suggested to us the name “*suelto*” (*suel* means “loose”). Small chromosomal deficiencies that separately uncover *sola* and *suel* phenocopy the respective RNAi effects (data not shown).

The live embryo RNAi assay also can be used to identify genes involved in muscle function. We found that the muscle pattern was entirely normal in embryos injected with CG2708 dsRNA, but these muscles never contracted when compared with age-matched control embryos (Video S1). CG2708 is expressed only in FCMs (Figure 4N–4R) and encodes a myosin-binding protein with homology to *Caenorhabditis elegans unc-45,* for which loss-of-function mutations are associated with muscle paralysis [46].

Finally, an RNAi phenotype was obtained for *chicadee (chic)* that encodes a *Drosophila* profilin homolog [47] that is expressed specifically in FCMs. RNAi for *chic* is associated with complete absence of cellularization at the blastoderm stage (data not shown), presumably due to dsRNA effects on both maternal and zygotic transcripts. Due to its maternal expression and essential involvement in oogenesis, it has not previously been possible to assess the early embryonic functions of *chic* using germline clonal analysis [48], underscoring another advantage of the RNAi approach used here.

## Discussion

We have used an integrated strategy for systematically studying the development of a complex tissue by combining genetic perturbations of a particular biological process, computational analysis of a compendium of gene expression profiles that is targeted to the tissue by FACS purification of the cells of interest, large-scale validation of predicted gene expression patterns by whole embryo in situ hybridization, and RNAi-based functional studies of newly discovered genes. Specifically, we identified large numbers of genes that are coexpressed in different subsets of myoblasts by analyzing pooled microarray data obtained for embryonic mesodermal cells purified from multiple genetic backgrounds in which muscle development is selectively perturbed. A whole embryo RNAi assay then revealed the developmental functions of selected myoblast-specific genes. Collectively, the present work contributes valuable information to a more detailed understanding of the regulatory network governing somatic myogenesis in the *Drosophila* embryo, provides a substantially expanded framework for future studies of this developmental process, and offers a unified experimental approach that can be applied to other systems.

### Transcriptional Profiling of Complex Tissues

Cell-specific genetic programs must be delineated in order to fully understand how diverse cellular identities are established during tissue and organ formation. Previous studies have addressed various aspects of metazoan development by combining genetic and genomic methods [9,14,27,28,49–55]. While highly informative for temporal aspects of gene expression in whole animals [50], in revealing sex-biased transcription [53], or in yielding cell-specific wild-type expression profiles [49,51,54,55], such studies have not examined the global changes in gene expression that are associated with genetic manipulations of regulatory pathways affecting the tissue of interest. Mutants that perturb large numbers of cells arising from subdomains of an embryonic axis have been used to enrich for the detection of tissue-specific transcripts, a strategy that works best for early aspects of development [14,27,28]. However, this genetic approach complicates the analysis of later steps in organogenesis since tissue organization and intercellular communication are severely disrupted by these major patterning mutations [28]. Perturbation of a single regulatory pathway in whole embryos has also been used for the discovery of cell-specific genes, but efforts like this have been limited by very high false-positive detection rates because the signal from the cells of interest is diluted by the rest of the embryo [52].

The present approach provides two major advantages for determining the gene expression programs of separate cell types in a developing embryo. First, isolating the tissue of interest—even without purifying individual cell populations—substantially increases the sensitivity of microarray experiments. Second, perturbation of multiple convergent pathways significantly augments both the statistical and biological power of the microarray compendium to resolve cell-specific expression patterns. While independent replicas of the same genotype yield statistical power, use of multiple genotypes has the additional benefit of reducing systematic biases that may be associated with a single genetic manipulation. Indeed, we found that different genotypes have distinct capacities to detect FC versus FCM genes, suggesting that perturbing multiple pathways is a more effective means to query diverse cell types present in the isolated tissue. For instance, the overall sensitivity of the approach is reflected in the high FC meta-analysis rank obtained for *eve* (108), even though it is expressed in less than 1% of mesodermal cells.

Purification of specific cells and the inclusion of multiple informative genotypes in the acquisition of genomewide expression data for a particular tissue—what we have termed a targeted expression profile compendium—provide additional information that has not been available from prior genomic studies of mesoderm development [14,27,28]. For example, a related microarray analysis of myoblast gene expression [28] predicted a total of only 33 FC and 48 FCM genes compared with 373 and 276, respectively, predicted here. Several important differences in experimental design can account for the disparate outcomes of the two approaches, including use of different numbers of genetic perturbations of FC and FCM development (two in the previous study versus 12 here), different microarray platforms representing dissimilar fractions of the genome, and the absence of Dpp as an FC determining signal in the embryos used in the earlier study [28]. In this regard, we found that Dpp contributes significantly to FC gene identification, so its inclusion in any experimental analysis of muscle development appears to be critical.

Our findings emphasize the importance of independently validating microarray data and computational predictions of genes expressed in different cell populations. Whereas whole embryo in situ hybridizations revealed that the FC gene prediction rate was very high, the fraction of true positive FCM genes was considerably smaller when the same datasets were analyzed using a similar rationale and statistical methods. The in situ hybridization results further demonstrated that the observed difference in the accuracy of FC and FCM gene prediction rates is largely attributable to an unanticipated number of genes expressed in both myoblast types that, from the microarray data analysis alone, were incorrectly scored as FCM-specific genes. This last outcome most likely occurred because transcripts expressed in both FCs and FCMs followed an FCM-specific pattern in the genetic perturbation and microarray experiments owing to the fact that FCMs greatly outnumber FCs in the purified cell fraction. This issue notwithstanding, the integrated approach we used facilitated the efficient identification of several hundred genes having different myoblast-specific expression patterns while entailing quite manageable false positive detection rates.

The transcriptional profiling strategy elaborated here offers an information-rich approach that can be applied to other model organisms and developmental processes. Indeed, because the present experiments employed a general mesodermal *Gal4* driver, the existing compendium of expression profiles should be applicable to mesodermal derivatives other than somatic muscle. Consistent with this expectation, a preliminary meta-analysis using a relevant subset of the present data was effective in predicting genes with cardiac expression (SEC and AMM, unpublished results). The sensitivity and specificity of these analyses can be further optimized by using the most appropriate combination of mutants, and by selectively targeting GFP for cell purification. Perhaps most important, the collective expression data obtained from such experiments provide vast amounts of information about the various regulatory inputs to each identified gene and allow detailed molecular signatures to be derived for specific cells within a complex tissue.

### Unanticipated Complexity of the *Drosophila* Muscle Regulatory Network

Muscle FCs are specified by the convergent inputs of multiple intercellular signals [10,17]. The differential expression of a few cell-specific markers has in the past suggested that individual FCs have distinct signaling responses, causing each to acquire a unique identity prior to its differentiation into a particular muscle. With the discovery of substantially more genes expressed in different FC subsets, the present work substantiates this hypothesis. Moreover, earlier studies anticipated that distinct but related transcriptional codes would be responsible for different patterns of FC gene expression [24,56]. This model is supported by recent computational and empirical analyses of candidate *cis*-regulatory modules associated with the FC genes newly identified here (A. A. Philippakis, B. Busser, S. S. Gisselbrecht, F. S. He, B. Estrada, A. M. Michelson, and M. L. Bulyk, unpublished data).

In contrast to FCs, the FCM population has been thought to be relatively homogeneous [15,16], an idea that is not supported by our findings. Rather, this second myoblast class is quite heterogeneous, and the control of FCM gene expression—while having some common features—is not uniform. For example, although transcription of most FCM genes requires *lmd,* others are entirely *lmd* independent. Still other FCM genes exhibit regional differences in their responses to perturbations of Ras and Notch signaling, while some *lmd*-dependent genes are not expressed in all FCMs in which Lmd is found. Finally, a subset of FCM genes is differentially controlled by Ras, Notch, and Lmd in the somatic and visceral subdivisions of the mesoderm, even though both types of muscle arise through fusion of similar myoblasts [57].

FCs and FCMs were found to have gene expression signatures comprising large numbers of unique genes, as well as numerous shared transcripts. Whereas transcription factors, signal transduction components, and adhesion molecules are overrepresented in FCs, proteins associated with metabolic functions predominate in both myoblast classes. The prominent expression of regulatory genes in FCs is in agreement with prior evidence that these myoblasts contain specific determinants of muscle identity [18,42] and suggests that cell fusion plays an important role in the acquisition of unique genetic programs by individual myotubes.

### Functions of Newly Identified Myoblast Genes

The specific functions of each myoblast type are further emphasized by our RNAi results. For example, *sola*—which encodes the *Drosophila* homolog of verprolin, an actin binding protein—is expressed only in FCMs and is essential for myoblast fusion. Moreover, profilin, another actin binding protein encoded by *chic* [48], is also restricted to FCMs. These findings imply a different function or mode of regulation of the actin cytoskeleton in FCMs as opposed to FCs during fusion. While the cytoskeleton has previously been implicated in myotube formation [58], an asymmetrically expressed cytoskeletal component has not been uncovered, further highlighting the unique nature of the cytoskeleton in these myoblasts. In contrast, RNAi directed against the FC-specific gene *suel*/CG17492 causes an early myospheroid phenotype in a subset of muscles, suggesting a defect in myotube pathfinding and/or in formation of stable epidermal attachments, functions characteristic of FCs [42].

Although whole-genome RNAi screens have proved to be highly informative for C. elegans and for cultured cells where efficient dsRNA delivery methods are available [59], they are technically much more difficult to apply to *Drosophila* embryos. Restricting a whole embryo RNAi screen to a list of genes having tissue-specific expression patterns offers a more efficient approach to such functional discovery. This concept can also be applied to large-scale RNAi analysis of mouse embryonic development.

The experimental strategy presented here has provided substantial insight into the complexity of components involved in muscle development in the *Drosophila* embryo. Many of our conclusions could only be drawn by examining the large, interrelated datasets that comprise a targeted expression profile compendium. Other findings are derived from more traditional studies of single genes that nevertheless depended on genomewide approaches for their identification. Further analysis of our existing results, and expansion of this database by performing similar experiments with additional informative genotypes and with smaller subsets of purified cells, should yield even greater knowledge of the architecture and function of the myogenic network. Furthermore, application of this integrated set of approaches in other developmental contexts, both in *Drosophila* and in other model organisms, can offer a systems-level view of cell fate specification and morphogenesis that provides a wealth of hypotheses for further testing by genetic and biochemical methods.

## Materials and Methods

### Fly strains and genetics.

The following *Drosophila* stocks were used to obtain both wild-type and genetically modified mesodermal cells expressing GFP: *twi*-*Gal4* UAS-2*EGFP* [30], UAS-λ*top* (constitutively activated EGFR) [60], UAS-*dof* UAS-λ-*htl* (constitutively activated Heartless FGFR together with Downstream of FGFR/Heartbroken/Stumps) [61,62], UAS-*Ras1^Act^* (activated Ras) [18], UAS-*pnt^P2VP16^* (activated Pointed) [24], UAS-*tkv^QD^* (activated Thick veins) [63], UAS-*arm^s10^* (activated Armadillo)[64], UAS-*arm^s10^;* UAS-*Ras1^Act^,* SG24 *wg^CX4^*/*CyO, wg^IG22^* UAS-2*EGFP,* UAS-*N^intra^* [65], *twi*-*Gal4 lmd^1^*/*TM3 ftz-lacZ,* UAS-2*EGFP lmd^2^/TM3 ftz-lacZ, twi*-*Gal4 Dl* ^X^/TM3 *ftz-lacZ,* and UAS-2*EGFP Dl* ^X^/*TM3 ftz-lacZ*. The following stocks were used to determine gene expression patterns in mutant backgrounds: *twi*-*Gal4,* UAS-*Ras1^Act^, Dl^X^*/*TM3 ftz-lacz,* and *lmd* ^1^/*TM3 ftz-lacz*. The enhancer trap line *rp298lacz* was used to test for localization of gene expression to founder cells [32].

### Fluorescence-activated sorting of cells from *Drosophila* embryos.

Freshly laid embryos were collected and aged to stage 11, at which point a single cell suspension was prepared. Cells were separated into GFP-positive and -negative cell populations using a flow cytometer (see Protocol S1 for details).

### Microarray experiments and data analysis.

Total cellular RNA (2.5 to 3 μg) was labeled in one round of linear amplification and used for hybridization to a single Affymetrix GeneChip using standard methods recommended by the manufacturer (http://www.affymetrix.com/support/technical/manual/expression_manual.affx). Each RNA sample was independently labeled and hybridized in triplicate. A detailed description of all computational methods used for analyzing the expression data can be found in Protocol S1.

### In situ hybridization and immunohistochemistry*.*

Digoxigenin-labeled antisense RNA probes were synthesized using cDNA clones obtained from the Drosophila Gene Collection (DGC1 and DGC2, http://www.fruitfly.org/DGC/index.html). For genes without an available cDNA, gene-specific PCR primers were designed. A microtiter plate method was used for parallel synthesis of multiple probes (http://www.fruitfly.org/about/methods/RNAinsitu.html). In calculating the true-positive detection rate for genes enriched in wild-type GFP-expressing cells, we considered as true positive every gene validated as having mesodermal expression by our in situ hybridizations or annotated as such in the BDGP in situ database or in the published literature (Table S1); a small number of genes were included in this GFP-positive category that were found to be expressed in nonmesodermal cells that nevertheless expressed GFP at stage 11 under *twi*-Gal4 control (for example, due to GFP perdurance in cells of the endodermal and mesectodermal primordia in which *twi* is expressed at earlier stages [unpublished data]). Antibody stainings were carried out as described [18] Rabbit anti-Lmd (from H. Nguyen) was used at 1:1,000. Homozygous *Dl* or *lmd* mutant embryos were identified using a *lacZ*-marked TM3 balancer chromosome.

### RNA interference assay.

Gene segments for dsRNA synthesis were selected to be 300 to 700 bp in length and common to all predicted splice variants of the targeted gene and to lack any consecutive 18 bp of identity to any other predicted gene. These sequences were PCR-amplified from primary embryonic cDNA using primers that incorporated T7 promoters on both ends (primer sequences are available upon request). Purified PCR product was transcribed in vitro and purified using the MEGAscript RNAi kit (Ambion, Austin, Texas, United States), precipitated, resuspended, and diluted to 2 mg/ml in DEPC-treated 1× injection buffer [40]. Dechorionated MHC-tau-GFP embryos [41] were injected mid-ventrally during the syncytial blastoderm stage, then allowed to develop to stage 16 to 17 before assessment. Each gene was initially injected and scored blindly, with negative control (*lacZ* dsRNA) and positive control (*mbc* or *blow* dsRNA) injections performed in parallel. Only embryos that developed robust GFP expression and lacked obvious major morphological defects (typically 60% to 80% of those injected) were included in the analysis.

## Supporting Information

Figure S1 Embryonic Expression Patterns of Selected Genes Identified in Microarray Experiments as Being Enriched in Wild-Type Mesoderm RNA in situ hybridization shows that validated mesodermally enriched genes are expressed in different populations of mesodermal cells at stage 11, including somatic and visceral muscle precursors (A, C, D–N, P and A, C–L, O, respectively), hemocytes (O), and cardiac primordium (D, E, I, and L–N). Arrowhead (I) indicates representative cardiac primordium; arrow (K) indicates visceral mesoderm; asterisk (O) indicates hemocytes.
(3.0 MB PDF)

Figure S2 Supporting Figures for FC and FCM Gene Meta-Analyses (A and C) Bar plot showing the weight of each genotype in the meta-analysis to identify genes with FC- and FCM-like expression, respectively (Protocol S1, Analysis E). Error bars show the standard deviation of weights within the approximately 2,000 weight profiles used to calculate each average weight profile.
(B and D) Normalized median absolute deviation between the meta-analysis gene rank (x-axis) and individual genotype ranks (Protocol S1, Analysis F). The graph shows the average over all the genotypes, using the weights in (A) and (C), respectively. The black vertical line highlights the point at which the data cross the trend line (blue) derived from a smoothing function (see Protocol S1, Analysis Method F).
(152 KB PDF)

Protocol S1 Experimental Procedures (93 KB DOC)

Table S1 Description of Mesodermally Enriched Genes; Validated FC, FCM, and FC + FCM Genes; and Results from In Situ Hybridization and RNAi Experiments (337 KB XLS)

Table S2 Meta-Analysis of the Transcriptional Profiling Data: Ranking of All Affymetrix Probe Sets by FC- or FCM-Like Gene Expression Pattern (5.9 MB XLS)

Table S3 Comparison of Top-Ranking FC and FCM Gene Lists in Terms of GO Functional Category Enrichment (48 KB XLS)

Video S1 Inactivation of the FCM Gene CG2708 by Injection of dsRNA Renders Embryos (right) Immotile When Compared with Age-Matched Embryos Injected with an Inactive Control dsRNA (left) Confocal images of GFP fluorescence were collected at 5-s intervals and presented at five frames per second.
(1.4 MB MOV)

### Accession Numbers

Microarray data described in the text are available from the Gene Expression Omnibus (www.ncbi.nlm.nih.gov/geo) with the accession number GSE3854.

Flybase (www.flybase.org) ID numbers for genes cited in the text are *eve,* FBgn0000606; *wg,* FBgn0004009; *dpp,* FBgn0000490; *Egfr,* FBgn0003731; *htl,* FBgn0010389; *Ras85D,* FBgn0003205; *N,* FBgn0004647; *lmd,* FBgn0039039; *Tl,* FBgn0003717; *twi,* FBgn0003900; *duf,* FBgn0028369; *Dl,* FBgn0000463; *CG13503,* FBgn0034695; *CG17492,* FBgn0032742; *CG2708,* FBgn0010812; *chic,* FBgn0000308; *dof,* FBgn0020299; *ftz,* FBgn0001077; *blow,* FBgn0004133; *CG14207,* FBgn0031037; *CG10275,* FBgn0032683; *CG10641,* FBgn0032731; *NHP2,* FBgn0029148; *RpI135,* FBgn0003278; *sns,* FBgn0024189; *GFP,* FBgn0014446; *Gal4,* FBgn0014445; and *lacZ,* FBgn0014447.

## Abbreviations

Dpp: Decapentaplegic
dsRNA: double-stranded RNA
EGF: epidermal growth factor
EGFR: epidermal growth factor receptor
FACS: fluorescence activated cell sorting
FC: founder cell
FCM: fusion-complement myoblast
FGF: fibroblast growth factor
FGFR: fibroblast growth factor receptor
GO: Gene Ontology
iRNA: in vivo RNA interference
RTK: receptor tyrosine kinase
Wg: Wingless
