# Supplementary material for: An Integrated Strategy for Analyzing the Unique Developmental Programs of Different Myoblast Subtypes
Source: PLoS Genet. 2006 Feb 17;2(2):e16. doi: 10.1371/journal.pgen.0020016 (PMC1366495; doi:10.1371/journal.pgen.0020016)
Supplement: Supplementary file 1 [file pgen.0020016.sg001.pdf]

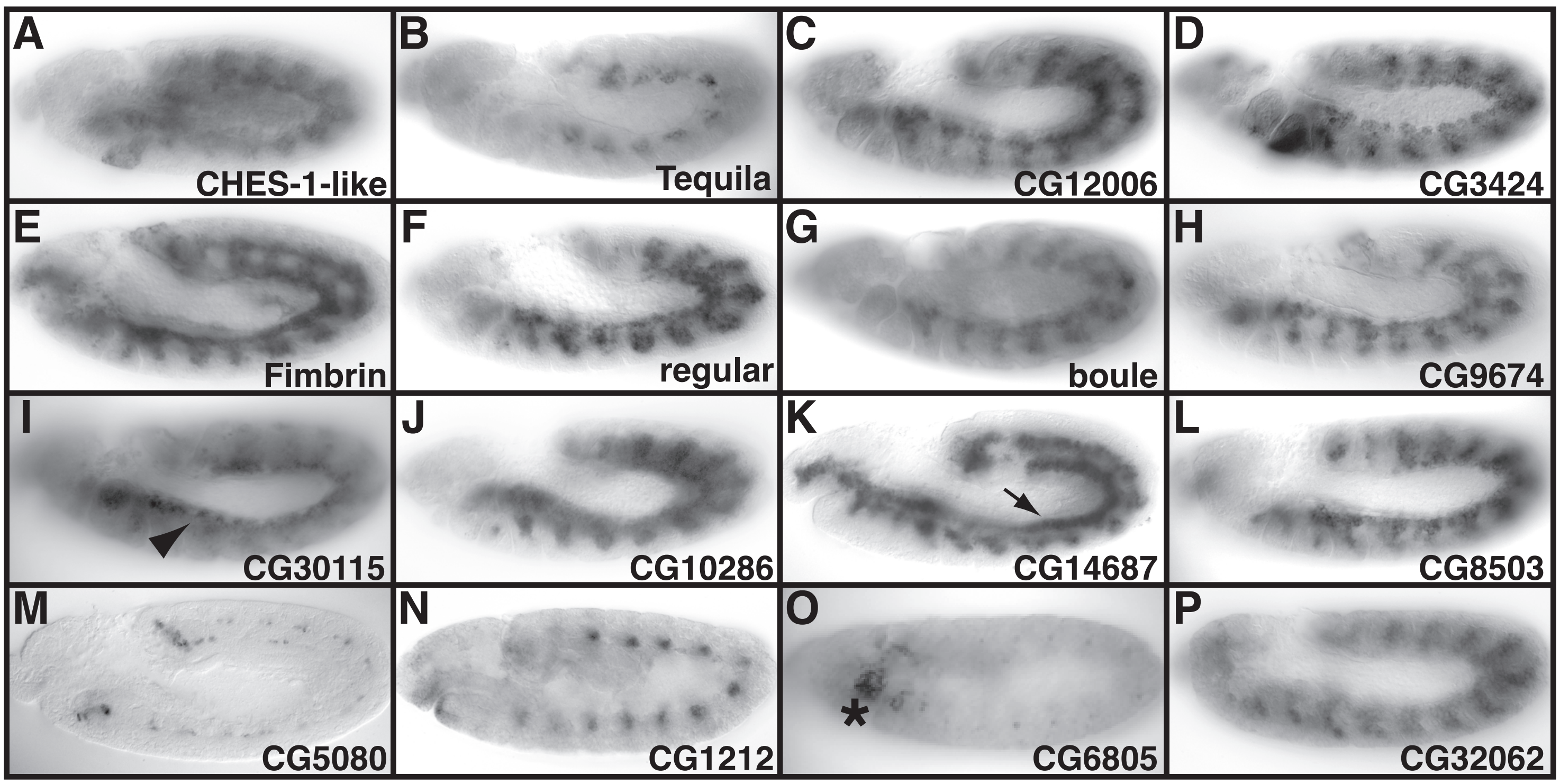
A
CHES-1-like
B
Tequila
C
CG12006
D
CG3424
E
Fimbrin
F
regular
G
boule
H
CG9674
I
CG30115
J
CG10286
K
CG14687
L
CG8503
M
CG5080
N
CG1212
O
CG6805
P
CG32062
