# Supplementary material for: An Integrated Strategy for Analyzing the Unique Developmental Programs of Different Myoblast Subtypes
Source: PLoS Genet. 2006 Feb 17;2(2):e16. doi: 10.1371/journal.pgen.0020016 (PMC1366495; doi:10.1371/journal.pgen.0020016)
Supplement: Supplementary file 2 [file pgen.0020016.sg002.pdf]

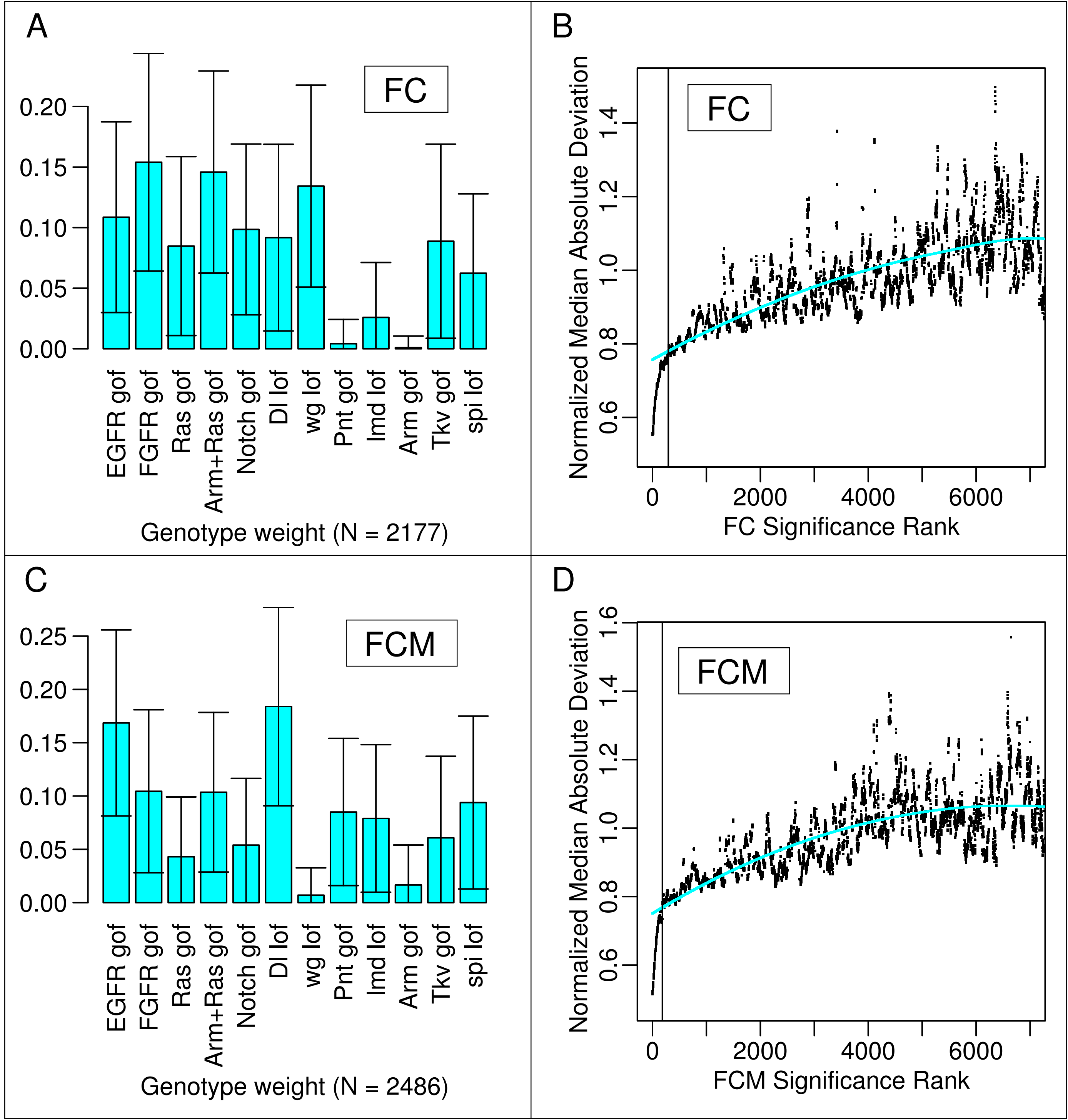

A
FC
0.00
0.05
0.10
0.15
0.20
EGFR gof
FGFR gof
Ras gof
Arm+Ras gof
Notch gof
Dl lof
wg lof
Pnt gof
Imd lof
Arm gof
Tkv gof
spi lof
Genotype weight (N = 2177)
B
FC
Normalized Median Absolute Deviation
0.6
0.8
1.0
1.2
1.4
0
2000
4000
6000
FC Significance Rank
C
FCM
0.00
0.05
0.10
0.15
0.20
0.25
EGFR gof
FGFR gof
Ras gof
Arm+Ras gof
Notch gof
Dl lof
wg lof
Pnt gof
Imd lof
Arm gof
Tkv gof
spi lof
Genotype weight (N = 2486)
D
FCM
Normalized Median Absolute Deviation
0.6
0.8
1.0
1.2
1.4
1.6
0
2000
4000
6000
FCM Significance Rank
