# Supplementary material for: An Integrated Strategy for Analyzing the Unique Developmental Programs of Different Myoblast Subtypes
Source: PLoS Genet. 2006 Feb 17;2(2):e16. doi: 10.1371/journal.pgen.0020016 (PMC1366495; doi:10.1371/journal.pgen.0020016)
Supplement: Supplementary file 3 [file pgen.0020016.sd001.doc]

**An Integrated Strategy for Analyzing the Unique Developmental Programs of**

**Different Myoblast Subtypes**

Beatriz Estrada, Sung E. Choe, Stephen Gisselbrecht, Sebastien Michaud, Lakshmi Raj,

Brian Busser, Marc S. Halfon, George M. Church and Alan M. Michelson

# Supplementary Experimental Procedures

# Computational analysis of expression profiling data

***Analysis Method A****. Initial method for identifying differentially expressed genes (applied to the mesodermal enrichment comparison).* Our expression profiling dataset consists of a series of comparisons between replicated experimental and control chips which have been processed in parallel. The first analysis step is to identify the differentially expressed genes (DEGs) for each comparison. Our initial strategy was to process the expression data using two different analysis methods, and then to combine the results so that the DEGs are less likely to be an artifact of any one analysis method. We chose two popular algorithms, dChip (using the “PM only” option, (Li and Wong, 2001) and RMA (robust multichip average, (Irizarry et al., 2003)) to convert the raw signal intensities into expression level summaries for each probe set. Each expression summary dataset was then filtered to eliminate probe sets with consistently low (background-level) signal. To find these probe sets, we calculated a detection p-value for each probe set, in each of the two samples being compared, by applying the Fisher method to combine the MAS 5.0 detection p-values from the replicate chips of each sample ([http://www.affymetrix.com](http://www.affymetrix.com/), and Analysis Method B). If the combined p-value for a given probe set is greater than a cutoff (0.05) for both control and experiment (i.e. called “absent” in both samples), then the probe set is filtered out. In a typical dataset, about 5000 (out of 14010) probe sets remain after applying the filter. The statistical significance of differential expression was assessed for each filtered set of expression summaries by applying the Significance Analysis of Microarrays Excel Add-in (Tusher et al., 2001), using the paired comparison option. In order to combine the two sets of results, the Fisher method was again applied, to obtain a summary estimate of significance that takes into account both the dChip and RMA results. For the mesodermal enrichment data, we applied a q-value cutoff of 0.05 to the minimum of the dChip, RMA, and combined q-values, and a fold-change cutoff of 1.39 (at first, the fold change cutoff was 1.5, but the false positive rate was sufficiently low that we decided to continue validating below fold change = 1.5).

***Analysis Method B****. Fisher method to combine p-values*. We used the Fisher method (Fisher, 1932) to combine the p-values from the replicate chips of each sample, obtaining a single detection p-value per sample. Briefly, for each probe set *i* and sample *j*, we calculate $S_{\text{ij}}\text{=-}2\sum_{k=1}^{n_{j}} \text{ln}(p_{\text{ijk}})$, where *k* is the *k*th replicate chip of sample *j*, and *n_j_* is the number of replicates of sample *j*. The combined p-value is determined from *S_ij_*, which follows a chi-square distribution with $2n_{j}$ degrees of freedom. In each case that the Fisher method was applied (Analysis Method A), directly combining the multiple sources of data would have been preferable, but was not possible due to technical limitations existing at that time.

***Analysis Method C****. New method for identifying DEGs, based on a control spike-in dataset (applied to the genotype-to-wild type comparisons)*. During the course of this study, we developed a control spike-in dataset which permits the relative assessment of different analysis methods. Studying this dataset led us to identify a set of analysis options that optimize DEG detection (Choe et al., 2004). We decided to process all the genotype-versus-wild-type data using this set of options, most of which are implemented in Bioconductor’s “affy” package (http://www.bioconductor.org). Briefly, the procedure involves generating 8 expression summary datasets, using the MAS background correction method, 4 different normalization algorithms (loess, invariant set, quantile and constant), the MAS 5.0 estimate of non-specific signal, and 2 different robust estimators (Tukey-Biweight and median polish). The 8 expression summary datasets are then used to generate a summary significance statistic that includes an intensity-dependent error model, derived from CyberT (Baldi and Long, 2001). Permutation tests result in false discovery rate estimates corresponding to various significance statistic cutoff levels. For more details, refer to (Choe et al., 2004).

***Analysis Method D****. Mapping probe set IDs to Flybase Release 3.1*. The Affymetrix DrosGenome1 GeneChip was designed against version 1 of the Drosophila Gene Collection (DGC). In order to have the most up-to-date assignments of probe set ID to gene ID, we BLASTed the probe set target sequences (http://www.affymetrix.com/products/arrays/specific/fly.affx) to the set of transcripts from Flybase Release 3.1. We collected BLAST hits with E-values ≤ 10^-30^. The top hit was selected as the assigned gene ID for each probe set.

***Analysis Method E****: Meta-analysis to identify candidate FC and FCM genes*. An important goal of this study is to identify new FC and FCM genes. In order to prioritize candidate FC/FCM genes for verification by *in situ* and gene reporter assays, we ranked the genes based on the concordance of their expression patterns with the canonical FC (or FCM) pattern expected from prior experimental observations (Figure 1B). Instead of choosing a R^2^-based fold change pattern-matching method, we selected a meta-analysis that quantifies the increased signal-to-noise resulting from having multiple independent experiments in this study (Box 1 in (Ghosh et al., 2003)). In Ghosh et al., the “combined significance statistic” (T^orig^) was designated to be a sum of the individual t-statistics from each genotype-to-wild-type comparison, with the expected direction of response factored in. Specifically, $T_{j}^{\text{orig}}=\sum_{i=1}^{n} d_{i}t_{\text{ij}}$, where $T_{j}^{\text{orig}}$ is the combined significance statistic for gene *j*, $d_{i}\in\left\{ 1,-1 \right\}$ is the expected direction of the fold change for genotype *i*, and *t_ij_* is the t-statistic for gene *j* in the genotype-*i* – to – wild-type comparison. By focusing on the direction and statistical significance of the expression response in each genotype, the T metric is better suited for this study than the commonly used pattern-matching correlation coefficient, which emphasizes the relative magnitude of fold changes. In our general experience with expression data, fold-change magnitudes are not as reproducible as t-statistics (Choe et al., 2004). Additionally, fold-change magnitudes are not accurately characterized in this biological system.

We added two custom modifications to T to accommodate various features of the input dataset, elaborated below. Specifically, we calculated $T_{j}=\sum_{i=1}^{n} w_{i}d_{i}\text{sign}(t_{\text{ij}})\text{min}(\left| t_{\text{ij}} \right|,t_{i}^{C})$, where T*_j_* is the combined significance statistic for gene *j*, $d_{i}\in\left\{ 1,-1 \right\}$ is the expected direction of the fold change for genotype *i*, *t_ij_* is the CyberT t-statistic for gene *j* in the genotype-*i* – to – wild-type comparison (Analysis Method C, above), $t_{i}^{C}$ is the minimum absolute value of the CyberT t-statistic for which the corresponding q-value ≤ 0.0001 for genotype *i*, and *w_i_* is the weight for genotype *i* (with $\sum_{i} w_{i}=1$). The first modification was to scale the contribution of each genotype to the T metric by a weight factor, which was determined by how well that genotype detects known FC or FCM genes (Figure 4A). This adaptation was designed to account for the fact that some genotypes may be more effective than others at detecting FC (or FCM) genes. For example, we know that the *lmd* loss-of-function genotype is informative for FCM genes, but not FC genes. Additionally, some genetic perturbations may extensively affect gene expression in mesodermal cell populations outside of FCs and FCMs, resulting in dilution and/or loss of the somatic FC/FCM signal. In order to determine the best set of weights for finding FC (or FCM) genes, we calculated T’s for all possible weight profiles $\left\{ w_{1},\text{.}\text{.}\text{.},w_{n} \right\}$ with the criteria $w_{i}\in\left\{ \text{0, 0}\text{.}\text{1, 0}\text{.}\text{2, }\ldots\text{, 1} \right\}$ and $\sum_{i} w_{i}=1$. For each weight profile, we noted how many known FC (or FCM) probe sets are detected at several q-value cutoffs (0.0001, 0.0005, 0.001, 0.005, 0.01). We found that for both FCs and FCMs, a large number of weighting schemes can yield the best detection of known genes at all q-value cutoffs. However, the best-performing weight sets have some common features: for FCs, the FGFR, Arm+Ras and wg genotypes have the largest weight, while lmd, Pnt, and Arm are down-weighted. For FCMs, the wg and Arm genotypes are de-emphasized, and Dl and EGFR are heavily weighted. For FCs and FCMs separately, we identified a set of about 2000 weight profiles that maximize the number of known genes detected at all of the q-value cutoffs simultaneously (Supplementary Figure 1). The average of these profiles was chosen as the optimized weight profile, used to calculate T*_j_* and thus rank the genes based on their FC-like or FCM-like expression. Formally, this optimization is not under-determined, since there are 12 adjustable parameters (genotype weights) and ~40 x 12 (known probe sets x genotypes) CyberT t-statistic datapoints.

The second modification of T*_j_* was introduced upon observing that a substantial number of high T scores were the result of one highly significant genotype vs. wild-type comparison, presumably because the distribution of observed t-statistics is heavy-tailed. In order to favor the probe sets where the predicted response occurs in multiple genotypes, we modified the calculation of T so that any one genotype cannot dominate the signal. A cap to each genotype’s contribution to T was enforced; $t_{i}^{C}$ is the minimum absolute value of the t-statistic for which the corresponding q-value ≤ 0.0001 for the comparison between genotype *i* and wild type. As a result, the T score was adapted to favor genes that show the expected response in multiple genotypes. Q-values (the fraction that is false positive, within a list of genes passing a given T-value cutoff) were estimated for all possible T-value cutoffs, by applying the permutation test described in (Ghosh et al., 2003).

***Analysis Method F****. Method to detect regions of concordance between two ranked lists.* Here, the goal was to choose a reasonable number of top-ranking genes from the FC and FCM meta-analyses, to serve as the initial candidates for in situ verification. We assume that if a region of the meta-analysis rank ordering contains genes that also rank similarly in constituent genotypes, then it is likely that this rank region contains true signal. To quantify this, for each 100-gene window of the meta-analysis rank ordering, from rank *i* to *i*+99, we found the ranks of these same genes in each individual genotype-to-wild type calculation ($r_{i}^{j}$, the rank within genotype *j* for the gene with meta-analysis rank *i*) and then observed how different these ranks were from $\left\{ i,\ldots,i+\text{99} \right\}$ by using the median absolute deviation ($\text{MAD}_{i,i+\text{99}}^{j}=\text{mad}\left\{ i,\ldots,i+\text{99},r_{i}^{j},\ldots,r_{i+\text{99}}^{j} \right\}$. We generated a null distribution of MADs by averaging the MADs from 100 random orderings of the 14010 probe sets on the Affy chip ($\text{MAD}_{i,i+\text{99}}^{R}$). Finally, we combined the genotypes using the same weights as in the meta-analysis, and divided by the null MAD distribution from the random data, to obtain what we call the normalized median absolute deviation: $\text{NMAD}_{i,i+\text{99}}=\frac{\sum_{j} w_{j}\text{MAD}_{i,i+\text{99}}^{j}}{\text{MAD}_{i,i+\text{99}}^{R}}$. Plots of this metric shows a region of low rank with lower NMAD (and therefore higher correlation with the individual genotype data), and steep slope. Within a fairly narrow transition range, there is a sharp leveling off of the slope, which probably represents the region where the individual genotypes start to lose concordance with each other and thus result in more random meta-analysis orderings. By applying a smoothing function (loess) to the data (blue line, Supplementary Figure 1B,D), and observing where the data first crosses above this trend line, we placed the transition region to be at *i* = 294 for the FC meta-analysis, and *i* = 184 for the FCM meta-analysis. Since we are looking at 100-gene windows, this would mean that the top 394 FC-like genes and 284 FCM-like genes have concordant signal with the individual genotypes; we choose these to be our initial list for FC/FCM gene candidates. At these ranks, the corresponding q-values are 0.0021 (FC) and 0.0017 (FCM), which are roughly in agreement with the conservative cutoffs recommended in (Choe et al., 2004).

To approximate the actual number of FC-specific genes, we estimated the true positive detection rate (55%) and false negative rate (32%) for the top 373 candidate FC gene list. The true positive rate was derived by performing in situ hybridizations on 213 genes randomly chosen from the top 394 probe sets (representing 373 distinct gene candidates), and observing that 118 out of the 213 (55%) were indeed FC genes. From these figures, we estimate that there are about 321 authentic FC genes from the top 373 candidates: 23 (from the training set) + 118 (verified by experiment) + 55% x 137 (fraction of untested genes predicted FCs). To approximate the false negative rate, we noted that 23 of the original training set of 34 FC genes were detected within the top 373 FC candidates (Supplementary Table 2), leaving 11/34 = 32% undetected. Thus, the true number of FC genes can be estimated at 321 (= 217 x 34/23). At present, we have documented 160 validated FC genes, 29 from the literature, and 131 from the current study (Supplementary Table 2).

To approximate the actual number of FCM-specific genes, we estimated the true positive detection rate (15%) and false negative rate (24%) for the top 284 candidate FCM probe set list (containing 276 unique genes). The true positive rate was derived by performing in situ hybridizations on 123 genes randomly chosen from the top 276, and observing that 18 out of the 123 (15%) were indeed FCM genes. Thus, we estimate that 62 (=25 + 18 + 128 x (15%)) authentic FCM genes exist among the top 276 FCM candidates. To approximate the false negative rate, we noted that 25 of the original training set of 33 FCM genes were detected within the top 276 FCM candidates (Figure 4A and Supplementary Figure 1B), leaving 8/33 = 24% undetected. Thus, the true number of FCM genes can be estimated at 82 (= 62 x 33/25). At present, we have documented 51 validated FCM genes, 6 from the literature, 45 from the current study (Supplementary Table 2).

***Analysis Method G****. GO category enrichment between gene lists*. We wished to learn whether the validated FC, FCM and FC+FCM gene lists were different in terms of functional category annotations. To do this, we applied two approaches: the hypergeometric distribution for each of the three lists (FC, FCM and FC+FCM) individually, and GoSurfer (http://biosun1.harvard.edu/complab/gosurfer/) to compare between each possible pair of lists. The hypergeometric distribution allowed to estimate whether a gene list contained significant enrichment of a given GO category annotation. We used the “GOstats” package within Bioconductor ([http://www.bioconductor.org](http://www.bioconductor.org/)) to calculate p-values using the hypergeometric distribution, and then obtained false discovery rates (to adjust for multiple hypothesis testing), using the Benjamini-Hochberg correction method implemented in Bioconductor’s “multtest” package. The criteria for enrichment were FDR < 0.1, and at least 5 genes from the GO category must exist in the gene list. The GoSurfer application detects the relative enrichment with Gene Ontology (GO) terms as calculated by a modified Pearson’s chi-squared test, followed by a p-value cutoff of 0.20. Supplementary Table 3 details the categories that were differently enriched within and between the FC, FCM, and FC+FCM gene lists.

***Analysis H****. Filtering and clustering of the expression compendium*. To identify possible alternative expression patterns of interest, we filtered and clustered the expression profile dataset. First, we filtered for probe sets that are likely to be differentially expressed in some way within the dataset. We employed a filter designed to be less stringent than selecting the differentially-expressed genes from each genotype-vs-wild type comparison. For example, if a gene were not significantly differentially expressed in any Ras-activating genotype alone, but significant when combining all of these genotypes together in a meta-analysis, it would make sense to include this gene in the clustergram. Therefore, we employed a variant of the meta-analysis (Analysis E) to include probe sets with significant differential expression when combining any subset of genotypes. Specifically, for every possible subset of the 12 genotypes, we calculated T-scores, using equal weightings of the absolute value of the CyberT t-statistics for those genotypes (for a two-sided test). A permutation test was used to generate q-value estimates, and all probe sets with q < 0.02 were included into the clustergram. This was repeated for each possible subset of the 12 genotypes, yielding 4603 genes total. Second, we applied the self-organizing map clustering algorithm to the expression patterns for these 4603 genes, using a 300x1 geometry, and viewed the results using available software (Eisen et al., 1998; Tamayo et al., 1999). FC and FCM subclusters were delineated by visual inspection.

**Preparation of embryonic cell suspensions and flow cytometry**

A single-cell suspension was prepared by a modification of a published procedure (Bernstein et al., 1978). Embryos were collected and aged at 25°C to 5.5-7.5 hours after egg deposition, dechorionated by incubation for 5 minutes in 50% bleach, and rinsed successively in 0.01% Triton X-100 and distilled water. No more than 30 mg of embryos were dissociated in a loose-fitting Dounce homogenizer (VWR: catalogue # 62400-620; clearance of 0.0035-0.005 inches) in 7 mL of Schneider medium. Seven vertical strokes of the pestle was optimal for balancing single cell yield and viability. Cells were kept on ice continuously once dissociated. Embryo suspensions were first centrifuged at 40XG for 5 minutes to pellet debris. The supernatant was then centrifuged at 380XG for 10 minutes to pellet cells, which were resuspended in 8% FBS in Schneider medium and sieved through 40μm nylon mesh.

The resulting single cell suspensions were FACS sorted according to standard protocols, with the following modifications: the running buffer used for Drosophila cells was Seecof saline (6 mM Na_2_HPO_4_, 3.67 mM KH_2_PO_4_, 106 mM NaCl, 26.8 mM KCl, 6.4 mM MgCl_2_, 2.25 mM CaCl_2_, pH 6.8); the machine was cooled to 4°C during sorting; collection was into 1.5 mL aliquots of RNAlater (Ambion), and cells were then stored in this solution on ice for up to 6 hours. Sorted cell suspensions were then diluted with Schneider medium so that RNAlater was no more than 20% of the total volume and centrifuged at 5000XG for 15 minutes. Cell pellets were resuspended in Trizol (Invitrogen) at the highest feasible concentration, to maximize RNA yield (typically 200-300 μL of Trizol for 2-5 million cells). The RNA was stored in this form at -80°C until all collections of a given genotype were prepared, then purified according to the manufacturer’s instructions and stored precipitated in isopropanol at -80°C until needed for hybridization.

A typical experiment involved the collection of 350-1500 mg (wet weight) of embryos from 7000-12,000 flies, the sorting of 1-3 X 10^7^ total cells from the initial suspension, and the isolation of 1.6-8 X 10^6^ GFP-positive cells. At least six independent cell collections were pooled for gene expression profiling of a given genotype. Approximately 1 μg of total RNA was obtained from 1 X 10^6^ cells.

References

Baldi, P., and Long, A. D. (2001). A Bayesian framework for the analysis of microarray expression data: regularized t -test and statistical inferences of gene changes. Bioinformatics *17*, 509-519.

Bernstein, S. I., Fyrberg, E. A., and Donady, J. J. (1978). Isolation and partial characterization of Drosophila myoblasts from primary cultures of embryonic cells. J Cell Biol *78*, 856-865.

Choe, S. E., Boutros, M., Michelson, A. M., Church, G. M., and Halfon, M. S. (2004). Preferred analysis methods for Affymetric GeneChips revealed by a wholly-defined control dataset. Genome Biol *in press.*

Eisen, M. B., Spellman, P. T., Brown, P. O., and Botstein, D. (1998). Cluster analysis and display of genome-wide expression patterns. Proc Natl Acad Sci USA *95*, 14863-14868.

Fisher, R. A. (1932). Statistical Methods for Research Workers, 4 edn (London, Oliver and Boyd).

Ghosh, D., Barette, T. R., Rhodes, D., and Chinnaiyan, A. M. (2003). Statistical issues and methods for meta-analysis of microarray data: a case study in prostate cancer. Funct Integr Genomics *3*, 180-188.

Irizarry, R. A., Bolstad, B. M., Collin, F., Cope, L. M., Hobbs, B., and Speed, T. P. (2003). Summaries of Affymetrix GeneChip probe level data. Nucleic Acids Res *31*, e15.

Li, C., and Wong, W. H. (2001). Model-based analysis of oligonucleotide arrays: expression index computation and outlier detection. Proc Natl Acad Sci U S A *98*, 31-36.

Tamayo, P., Slonim, D., Mesirov, J., Zhu, Q., Kitareewan, S., Dmitrovsky, E., Lander, E. S., and Golub, T. R. (1999). Interpreting patterns of gene expression with self-organizing maps: methods and application to hematopoietic differentiation. Proc Natl Acad Sci U S A *96*, 2907-2912.

Tusher, V. G., Tibshirani, R., and Chu, G. (2001). Significance analysis of microarrays applied to the ionizing radiation response. Proc Natl Acad Sci U S A *98*, 5116-5121.
